# Clinical course of preterm prelabor rupture of membranes in the era of prophylactic antibiotics

**DOI:** 10.1186/1756-0500-5-515

**Published:** 2012-09-22

**Authors:** Vorapong Phupong, Lalita Kulmala

**Affiliations:** 1 Department of Obstetrics and Gynecology, Faculty of Medicine, Chulalongkorn University, Rama IV Road, Pathumwan, Bangkok 10330, Thailand

**Keywords:** Preterm, Prelabor rupture of membranes, Latency, Prophylactic antibiotic, Corticosteroids

## Abstract

**Background:**

Preterm prelabor rupture of membrane (PPROM) causes maternal and neonatal complications. Prophylactic antiobiotics were used in the management of PPROM. The objectives of this retrospective study were to compare clinical course and outcome of PPROM managed expectantly with prophylactic antibiotics and antenatal corticosteroids with those without prophylactic antibiotics and antenatal corticosteroids.

**Results:**

A total of 170 cases of singleton pregnant women with gestational age between 28–34 weeks suffering from PROM during January 1998 to December 2009 were included; 119 cases received prophylactic antibiotics and antenatal corticosteroids while 51 cases did not received prophylactic antibiotics and antenatal corticosteroids. Median latency period in the study group was significantly longer than in the control group (89.8 vs. 24.3 hours, P < 0.001). The percentage of patients who did not deliver within 48 hours and within 7 days in the study group were also significantly higher than in control group (64.7 vs. 31.4%, P < 0.001 and 29.4 vs. 7.8%, P = 0.002, respectively). Maternal infectious morbidity was comparable between groups (17.6% vs. 13.7%, P = 0.52). Neonatal infectious morbidity was significantly lesser in study group than control group (21% vs. 35.3%, p = 0.04).

**Conclusions:**

Latency period of PPROM after using prophylactic antibiotics and antenatal corticosteroids increased while neonatal infectious morbidity was low. But maternal infectious morbidity was not increased. This retrospective study confirms the benefit of prophylactic antibiotics and antenatal corticosteroids in management of PPROM.

## Background

Prelabor or premature rupture of membranes is defined as rupture of membranes before onset of labor [1]. Preterm prelabor rupture of membrane (PPROM) is prelabor rupture of membranes that occurs before 37 weeks gestation. PPROM usually results in preterm birth and causes 1/3 of preterm birth. PPROM causes complications not only in the neonate but also in the mother [1]. These complications are more common in PPROM of less than 34 weeks gestation [2].

In the past, management of women with PPROM was expectant without any medications. Several studies reported that the latency period after PPROM was 1.5-4.6 days [3-5]. Fifty to ninety-three percent cases and 69.3-97.3% cases delivered within 48 hours and 7 days following rupture of membranes, respectively [3-7].

After two large randomized controlled trials, prolongation of latency period is believed to reduce neonatal complications [8,9]. Thus, the American College of Obstetricians and Gynecologists (ACOG) prepares clinical management guidelines and recommends using prophylactic antibiotics in the expectant management of PPROM to prolong pregnancy, reduce maternal infectious morbidity and reduce infectious and gestational age-dependent neonatal morbidity. Corticosteroids are recommended to administer in PPROM to reduce the risks of neonatal prematurity related complications [10,11].

There have been a few reports of the clinical course and outcome of PPROM after using prophylactic antibiotics and antenatal corticosteroids. Therefore we retrospectively reviewed and compared the latency period, maternal and neonatal morbidity and mortality in patients with PPROM managed expectantly with prophylactic antibiotics and antenatal corticosteroids and without prophylactic antibiotics and antenatal corticosteroids.

## Methods

This study was approved by the Research Ethics Committee of the Faculty of Medicine, Chulalongkorn University, Bangkok, Thailand. Medical records of pregnant women with gestational age (GA) between 28–34 complete weeks with PROM admitted to the Department of Obstetrics and Gynecology, Faculty of Medicine, Chulalongkorn University, Bangkok, Thailand between January 1997 and December 2009 were reviewed. Singleton pregnancies with cervical dilatation less than 4 cm. at admission were reviewed. From year 1998–2009, only women who received prophylactic antibiotics to prolong latency period and had a single course of antenatal corticosteroids to induce fetal lung maturity were included into study group. Two-day’s intravenous ampicillin and oral macrolide (erythromycin, roxithromycin or azithromycin) followed by five days oral amoxicillin and oral macrolide (erythromycin, roxithromycin or azithromycin) were used as prophylactic antibiotics. In year 1997, women who were managed expectantly without prophylactic antibiotics and antenatal corticosteroids were included as a control group. We excluded cases that had fetal anomaly or any conditions that would require the pregnancy to be terminated upon admission including chorioamnionitis or fetal distress. Cases that received tocolysis were also excluded. Diagnosis of PPROM was based on a combination of history, gross leakage of amniotic fluid, ferning test, nile blue test and oligohydramnios by ultrasonogram. Latency period was defined as the time interval between the rupture of membranes and delivery. Omphalitis was defined as a localized infection of the umbilical cord stump [12].

Data on maternal age, gravidity, parity, total number of antenatal care visits, GA at admission, latency period, GA at delivery, mode of delivery, maternal and neonatal outcomes, and duration of hospitalization were extracted from the medical records of the patients. Demographic characteristics, latency period, maternal and neonatal outcomes were compared between groups.

### Statistical analysis

Statistical analysis was performed with SPSS software package version 12.0 (SPSS Inc, Chicago, IL, USA). Data was presented as mean ± standard deviation (SD), median and percentage. Continuous variables were compared by student t test and Mann–Whitney U test while chi square test, or Fisher exact test when appropriate, was used to compare categorical variables. A *P*-value < 0.05 was considered statistically significant.

## Results

A total of 170 cases of PPROM were included; 119 cases received prophylactic antibiotics and antenatal corticosteroids (study group) while 51 cases did not received prophylactic antibiotics and antenatal corticosteroids (control group). Demographic characteristics are shown in Table 1. There were no statistical differences between study and control groups for age, parity, total number of antenatal care visits and GA at admission.

**Table 1:** Demographic characteristics between groups

**Characteristics**	**Study group**	**Control group**	***P *value**
	**(n = 119)**	**(n = 51)**	
**Age**	26.8 ± 6.1	26.2 ± 5.4	0.54
**Parity**			0.91
** 0**	76 (63.9%)	33 (64.7%)	
** ≥1**	43 (36.1%)	18 (35.3%)	
**Total number of antenatal care visits**	5.1 ± 2.5	4.8 ± 0.6	0.39
**GA at admission (weeks)**	31.7 ± 1.6	31.8 ± 2.2	0.74
**Median (interquartile) GA at admission (weeks)**	32 (31, 33)	32 (30, 34)	0.22*

Data presented as mean ± SD or n (%).

*GA*: gestational age.

* Mann–Whitney U test was used.

Clinical course and maternal morbidity are shown in Table 2. Median latency period in the study group was significantly longer than in control group (89.8 vs. 24.3 hours, P < 0.001). The percentage of patients who did not deliver within 48 hours and within 7 days in the study group were also significantly higher than in control group (64.7% vs. 31.4%, P < 0.001 and 29.4% vs. 7.8%, P = 0.002, respectively). The number of pregnancy that reached 34 weeks were 31 (26.1%) and 15 (29.4%) in the study and control group, respectively (P = 0.07). And the number of pregnancy that reached 37 weeks were 1 (0.8%) and 0 (0%) in the study and control group, respectively (P = 1.00). GA at delivery was comparable. Median day of hospitalization in study group was significantly longer than in control group. Maternal infectious morbidity such as chorioamnionitis, postpartum metritis and surgical wound infections were comparable between groups. There was no maternal death in both groups.

**Table 2:** Clinical course and maternal morbidity between groups

	**Study group (n = 119)**	**Control group (n = 51)**	**P value**
**Latency periods** (hours) median (interquartile)	89.8 (26, 175.3)	24.3 (8.4, 60.3)	< 0.001
> 2 days	77 (64.7%)	16 (31.4%)	< 0.001
> 7 days	35 (29.4%)	4 (7.8%)	0.002
**GA at delivery (weeks)**	32.5 ± 1.9	31.9 ± 2.2	0.80
**Mode of delivery**			0.30
** - Vaginal delivery**	82 (68.9%)	31 (60.8%)	
** - Forceps & vacuum extraction**	3 (2.5%)	1 (2%)	
** - Cesarean section**	31 (26.1%)	15 (29.4%)	
** - Breech assisting**	3 (2.5%)	4 (7.8%)	
**Maternal infectious morbidity**	21 (17.6%)	7 (13.7%)	0.52
- Chorioamnionitis	19	6	
- Metritis	1	1	
- Wound infection	1	0	
**Median day of hospitalization** (interquartile)	8 (5, 12)	4 (2, 6)	< 0.001

Data presented as median (interquartile), mean ± SD or n (%).

*GA*: gestational age.

Neonatal outcome is shown in Table 3. There were no significant differences between study and control groups for sex, birth weight, Apgar scores, duration of neonatal hospitalization, neonatal intensive care unit (NICU) stay, and ventilation assistance. Rates of respiratory distress syndrome (RDS), intraventricular hemorrhage (IVH), necrotizing enterocolitis (NEC) and neonatal mortality were not significantly different between both groups. Neonatal infectious morbidity including sepsis, pneumonia, meningitis, and omphalitis were significantly less in the study group than in control group (21% vs. 35.3%, p = 0.04).

**Table 3:** Neonatal outcomes between groups

**Character**	**Study group (n = 119)**	**Control group (n = 51)**	**P value**
**Sex**			0.29
** - Male**	80 (67.2%)	30 (58.8%)	
** - Female**	39 (32.8%)	21 (41.2%)	
**Birth weight (grams)**	1944.7 ± 427.4	1896.3 ± 419.8	0.49
**Apgar scores**			
At 1 minute < 7	22 (18.5%)	8 (15.7%)	0.83
At 5 minutes < 7	5 (4.2%)	2 (3.9%)	0.58
**Median day of**	8 (5,20)	8 (5, 22)	0.69
**hospitalization** (interquartile)			
**RDS**	18 (15.1%)	6 (11.8%)	0.73
**IVH**	2 (1.7%)	1 (2%)	1.00
**NEC**	3 (2.5%)	2 (3.9%)	0.63
**Infectious morbidity**	25 (21%)	18 (35.3%)	0.04
** - Sepsis**	10	7	
** - Pneumonia**	9	9	
** - Meningitis**	2	1	
** - Omphalitis**	4	1	
**NICU stay**	38 (31.9%)	16 (31.4%)	0.94
**Ventilation assistance**	16 (13.4%)	13 (25.5%)	0.05
**Mortality**	2 (1.7%)	2 (3.9%)	0.58

Data presented as mean ± SD, median (interquartile) or n (%).

*RDS*: respiratory distress syndrome, *IVH*: intraventricular hemorrhage, *NEC*: necrotizing enterocolitis, *NICU*: neonatal intensive care unit.

## Discussion

This retrospective study compared clinical course and outcome of PPROM cases managed expectantly with prophylactic antibiotics and antenatal corticosteroids with those without prophylactic antibiotics and antenatal corticosteroids. Latency period was significantly longer in PPROM cases managed expectantly with prophylactic antibiotics and antenatal corticosteroids. Neonatal infectious morbidity was significantly lesser in cases managed expectantly with prophylactic antibiotics and antenatal corticosteroids. But maternal infectious morbidity was comparable. This finding supports the benefit of prophylactic antibiotics and antenatal corticosteroids in PPROM as shown in previous studies [8,9].

Since 1998, cases diagnosed as PPROM in our institute were managed expectantly as per recommendation from ACOG [10,11]. A 7-day course of parenteral ampicillin and oral therapy with amoxicillin and erythromycin was used during expectant management of PPROM hoping to prolong pregnancy and to reduce infectious and gestational age-dependent neonatal morbidity [9-11].

There have been several studies evaluating prophylactic antibiotics in PPROM [13-15]. Magwali TL et al. used co-amoxiclav in their study and found that antibiotics could prolong latency period and decreased neonatal and maternal morbidity due to sepsis [13]. Ryo E et al. used imipenem/cilastatin sodium in their study and found that imipenem/cilastatin sodium could prolong the latency period [14]. August Fuhr N et al. used mezlozillin in their study and found that antibiotics could prolong latency period and reduced neonatal infectious morbidity [15]. However, there has been no study evaluating the outcome of PPROM after the recommendation to use prophylactic ampicillin and erythromycin.

The usual outcome of PPROM is labor. The latency period was longer in the study group than in the control group (3.7 vs. 1.0 days, P < 0.001) which was similar to previously studies [8,9]. Mercer et al. performed a RCT study evaluating intravenous ampicillin and erythromycin for 48 hours followed by oral amoxicillin and erythromycin base for 5 days vs. placebo in PPROM. They found that antibiotics in the PPROM group had a longer median time to delivery than the placebo group (6.1 vs. 2.9 days, P < 0.001) [9]. Kenyon et al. performed a RCT evaluating erythromycin, co-amoxiclav, both, or placebo given four times daily for 10 days or until delivery. They found that the use of erythromycin was associated with prolongation of pregnancy in PPROM [8]. This suggests that antibiotics can suppress or prevent clinically significant intrauterine infection and shorten latency.

In present study, we found a significant increased number of patients who did not deliver within 48 hours and 7 days in study group when compared with a control group (64.7% vs. 31.4%, P < 0.001 and 29.4% vs. 7.8%, P = 0.002, respectively). This was similar to previous reports [8,9]. Mercer et al. found a significantly decreased number of women assigned to antibiotics compared with placebo delivered within 48 hours (27.3% vs. 36.6%, P = 0.03) and delivered within 7 days (55.5% vs. 73.5%, P = 0.001) [9]. Kenyon et al. demonstrated significantly fewer women on erythromycin alone delivered within 48 hours than did those on placebo (30.5% vs. 40.7%, P < 0.0001) [8].

The maternal infectious morbidity such as chorioamnionitis, metritis and wound infection was not different between groups in the present study. This is in contrast to previous studies [8,9]. Mercer et al. [9] found that the antibiotic group had a lower incidence of clinical amnionitis when compared with placebo (23.0% vs. 32.5%; P = 0.01). But, the incidence of postpartum endometritis was similar regardless of antibiotic treatment (11.0% vs. 11.5%; P = 0.85). Kenyon et al. [8] found that the use of both erythromycin and co-amoxiclav (5.0% vs. 8.4%, P = 0.001) and any antibiotic (6.2% vs. 8.4%, P = 0.008) was associated with significantly less uterine infection than use of placebo.

Neonatal infectious morbidity was significantly decreased in the antibiotic group in the present study. Mercer et al. found that maternal antibiotic therapy was associated with reductions in the incidence of neonatal pneumonia and sepsis in the group B streptococcus negative cohort [9]. A broad spectrum of aerobic and anaerobic bacteria and mycoplasmas have been implicated as causative agents for intrauterine infection in PPROM [16,17]. Thus, broad spectrum antibiotics (ampicillin plus erythromycin) are more beneficial in this condition.

The limitation of this study was retrospective study. We cannot control the confounding factors. Thus, bias may have been introduced. However, the demographic characteristics between groups in present study were not different. Further RCT comparing different regimens of antibiotics should be conducted.

## Conclusions

Latency period of PPROM after using prophylactic antibiotics and antenatal corticosteroids increased while neonatal infectious morbidity was low. But maternal infectious morbidity was not increased. This study confirms the benefit of prophylactic antibiotics and antenatal corticosteroids in management of PPROM.

## Competing interests

The authors declare that they have no competing interests.

## Authors’ contributions

VP conceived, carried out experiments and analysed data. LK carried out experiments. All authors were involved in writing the paper and had final approval of the submitted and published versions.
